# Supplementary material for: Regulation of Heat Stress in Physcomitrium (Physcomitrella) patens Provides Novel Insight into the Functions of Plant RNase H1s
Source: Int J Mol Sci. 2022 Aug 17;23(16):9270. doi: 10.3390/ijms23169270 (PMC9409398; doi:10.3390/ijms23169270)
Supplement: Supplementary file 1 [file ijms-23-09270-s001.zip › ijms-1790384-supplementary.pdf]

Table S1. Primers used for this article.

Primer name	Primer sequence (5'-3')	Usage
PpOG1-*A*-rec-F	CCAGTCACTATGGCGGCCGCATGGCGCCGAAGGCCAAAC	Overexpression of *A*
PpOG1-*A*-rec-R	TTCTCCTTTACCCATCTCGAGGTCTTCAATCTTTATCGAAATTG	Overexpression of *A*
F	CTTGGTGTTTCGAGCTTTTCC	PCR for *A*
R	CCATGATGTATACGTTGTGGG	PCR for *A*
Ppactin_F	CGGAGAGGAAGTACAGTGTGTG	qRT-PCR for actin
Ppactin_R	ACCAGCCGTTAGAATTGAGCC	qRT-PCR for actin
PpRNH1A-qPCR-F	ACCGGGCTTTCATATTAGGGT	qRT-PCR for *A*
PpRNH1A-qPCR-R	GGAGTCCTTCATCGTTCACC	qRT-PCR for *A*

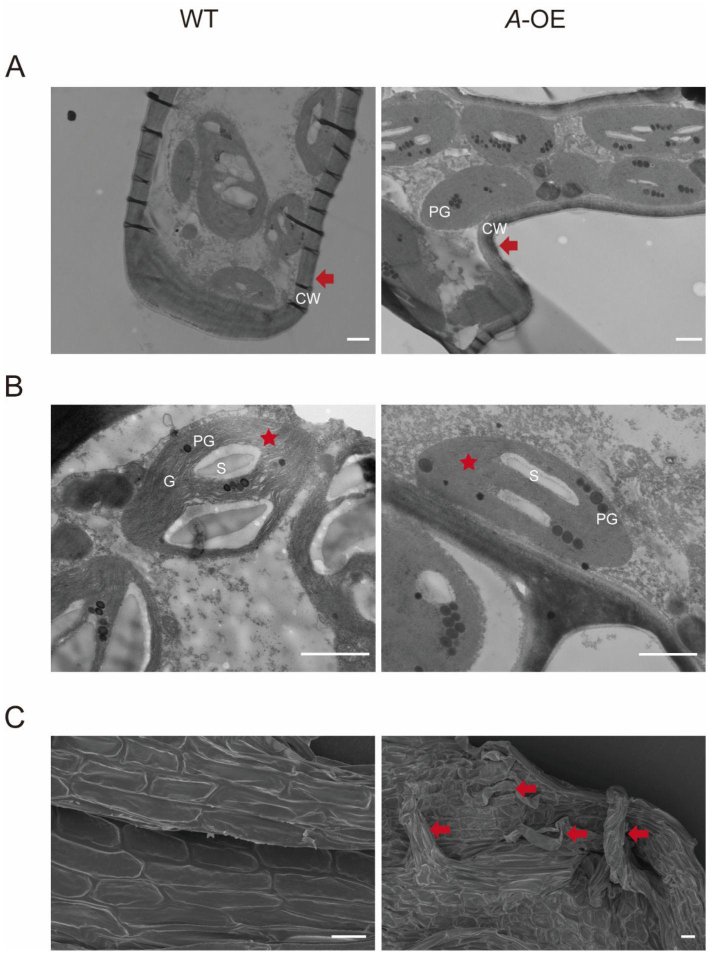

Figure S1. Morphology comparision between WT and *A*-OE plants. (**A**) Ultrastructure of cells of WT and *A*-OE were observed under transmission electron microscopy (TEM). Scale bar = 1 μm. CW: cell wall; PG: plastoglobuli; the red arrows indicate the difference in cell wall between the WT and *A*-OE plants. (**B**) Ultrastructure of chloroplasts of WT and *A*-OE were observed under transmission electron microscopy (TEM). Scale bar=1μm. S: starch; G: granum; the red stars indicate the difference in chloroplast structure between the WT and *A*-OE plants. (**C**) The epidermal structure of WT and *A*-OE were observed under the scanning electron microscope (SEM). The red arrows indicate the protrusions and abnormalities on the surface of the *A*-OE plants. Scale bar = 20 μm.
